# Increasing medium chain fatty acids production in *Yarrowia lipolytica* by metabolic engineering

**DOI:** 10.1186/s12934-018-0989-5

**Published:** 2018-09-10

**Authors:** Coraline Rigouin, Christian Croux, Vinciane Borsenberger, Maher Ben Khaled, Thierry Chardot, Alain Marty, Florence Bordes

**Affiliations:** 1 LISBP, Université de Toulouse, CNRS, INRA, INSA, Toulouse, France; 2 INRA, UMR1318, Institut Jean-Pierre Bourgin, Saclay Plant Sciences, Versailles, France; 3 AgroParisTech, Institut Jean-Pierre Bourgin, Saclay Plant Sciences, Versailles, France

**Keywords:** *Yarrowia lipolytica*, Elongase, Medium chain fatty acids, Fatty acid synthase, Diglyceride acyltransferase

## Abstract

**Background:**

Oleaginous yeast *Yarrowia lipolytica* is an organism of choice for the development of biofuel and oleochemicals. It has become a chassis for metabolic engineering in order to produce targeted lipids. Understanding the function of key-enzymes involved in lipid metabolism is essential to design better routes for enhanced lipid production and for strains producing lipids of interest. Because medium chain fatty acids (MCFA) are valuable compounds for biokerosene production, we previously generated strains capable of producing MCFA up to 12% of total lipid content (Rigouin et al. in ACS Synth Biol 6:1870–1879, 2017). In order to improve accumulation and content of C14 fatty acid (FA), the elongation, degradation and accumulation of these MCFA in *Yarrowia lipolytica* were studied.

**Results:**

We brought evidence of the role of YALI0F0654 (*Yl*ELO1) protein in the elongation of exogenous or de novo synthesized C14 FA into C16 FA and C18 FA. *Yl*ELO1 deletion into a αFAS_I_1220_W expressing strain leads to the sole production of C14 FA. However, because this strain does not provide the FA essential for its growth, it requires being cultivated with essential fatty acids and C14 FA yield is limited. To promote MCFA accumulation in *Y. lipolytica* without compromising the growth, we overexpressed a plant diglyceride acyltransferase specific for MCFA and reached an accumulation of MCFA up to 45% of total lipid content.

**Conclusion:**

We characterized the role of *Yl*ELO1 in *Y. lipolytica* by proving its involvement in Medium chain fatty acids elongation. We showed that MCFA content can be increased in *Yarrowia lipolytica* by promoting their accumulation into a stable storage form (triacylglycerides) to limit their elongation and their degradation.

**Electronic supplementary material:**

The online version of this article (10.1186/s12934-018-0989-5) contains supplementary material, which is available to authorized users.

## Background

Microbial oils are promising alternatives to fossil fuels [1]. In this context, there is a growing interest in microbe engineering to produce valuable fatty acids to be used as biofuel or oleochemicals [2–4]. Oleaginous organisms have been intensively explored for this production as they can accumulate high amount of lipids (storage capacity) and grow on various substrate (lipogenesis capacity) [5, 6]. Among the lipid-producer microorganisms, the yeast *Yarrowia lipolytica*, a GRAS organism (Generally Recognized As Safe), with considerable potential in industrial biotechnology [7, 8], is capable of producing and accumulating lipids up to 50% of its dry weight in large-scale fermentations [9]. Thanks to the recent advances in the development of genetic tools for *Y. lipolytica* [10, 11], this yeast has become the model of choice for the engineering applied to lipid metabolism, enabling enhanced accumulation [12–14] and the synthesis of specific lipids: Polyunsaturated fatty acids (PUFA) [15], fatty acid methyl ester (FAME) [16], fatty acid alcohol [17], free fatty acids (FFA) [18] and short or medium chain fatty acids (MCFA) [16, 19, 20]. Remarkably, MCFA are valuable compounds of the biofuel industry since kerosene is essentially composed of saturated FA with medium chain length FA [21].

Cellular fatty acids are derived from three different sources: endogenous lipid turnover, de novo synthesis and elongation, and external supply. In yeast, the cytosolic fatty acid synthase (FAS) catalyzes de novo fatty acids synthesis. The protein is composed of two subunits: αFAS consists of the acyl carrier protein (ACP), ketoacyl reductase (KR), ketoacyl synthase (KS) and phospho panthetheine transferase (PPT) domains, and βFAS consists of the acyl transferase (AT), enoyl reductase (ER), dehydratase (DH) and malonyl palmitoyl transferase (MPT) domains. The FAS is organized as a hexameric α6β6 complex and forms a giant multifunctional protein of 2.6 MDa. Each monomer encloses the eight functional domains that catalyze all the reactions required for fatty acid (FA) synthesis from acetyl-CoA and malonyl-CoA: activation, priming, multiple cycles of elongation and termination [22]. In yeast, the products consist mainly of C16 and C18 FA [23] to conform the physiological requirements of biological membrane. Very long chain fatty acids (VLCFA) synthesis occurs in the endoplasmic reticulum membrane and corresponds to the elongation of C16-C18 FA to C20-C26 FA [23]. The elongation follows similar reaction schemes as cytosolic FAS but functions as four distinct enzymatic reactions: condensation of the acyl chain with malonyl-CoA to form an elongated 3-ketoacyl-CoA catalyzed by ELO enzymes, then reduction to enoyl-CoA, followed by dehydratation and a second reduction to acyl-CoA [24]. In *Saccharomyces cerevisiae*, the VLCFA are incorporated into ceramide and serve as components of sphingolipids [25] and glycosylphosphatidylinositol-anchor lipid fractions [26]. They play an essential role in the cells as the deletion of one of the gene involved in their synthesis is associated with pleiotropic deficiencies.

The ketoacyl synthase (KS) domain of the cytosolic FAS complex and the elongase enzyme (ELO) of the ER membrane catalyze the first step of FA elongation. KS and ELO both ensure the same condensing function but there is no sequence or predicted topological similarity between ELO and KS families. KS mechanism for C–C bond formation is a decarboxylating Claisen condensation [27]: the enzyme displays the catalytic triad Cys, His, His/Asn allowing the acyl-CoA to covalently bind to the active site cysteine. Mechanism of ELO proteins has not been elucidated yet, there is no conserved Claisen-like condensing enzyme catalytic triad but instead, signature sequence motifs [28] including an histidine box, a motif found in enzyme carrying out oxidative reactions [29].

We and another team recently showed that mutations in the KS domain of the yeast FAS lead to change in fatty acid composition [19, 30]. In *Y. lipolytica* KS domain, when the active site Isoleucine 1220 residue is replaced by an aromatic residue (i.e. tryptophan), a steric clash occurs and prevents the accommodation of long chain fatty acids [19]. Consequently, the fatty acid profile of the resulting strain shifts toward shorter chains allowing for the accumulation of C14 FA up to 11.6% of total lipid content during stationary phase. However, we hypothesized that elongation may occur upon FA de novo synthesis and that the actual FAS MCFA products may be underestimated. Two protein sequences of potential elongases (YALI0B20196p and YALI0F06754p) have been identified in *Y. lipolytica* [31] but their function have not been elucidated yet.

In this paper, the main objective was to enhance MCFA production in *Y. lipolytica*. We investigated the role of ELO proteins in *Y. lipolytica* to highlight a potential function in the elongation of medium chain fatty acids and we studied the impact of ELO deletion in a mutant strain capable of producing MCFA.

## Results

### Identification of elongase sequences in *Y. lipolytica* proteome

**Fig. 1:**
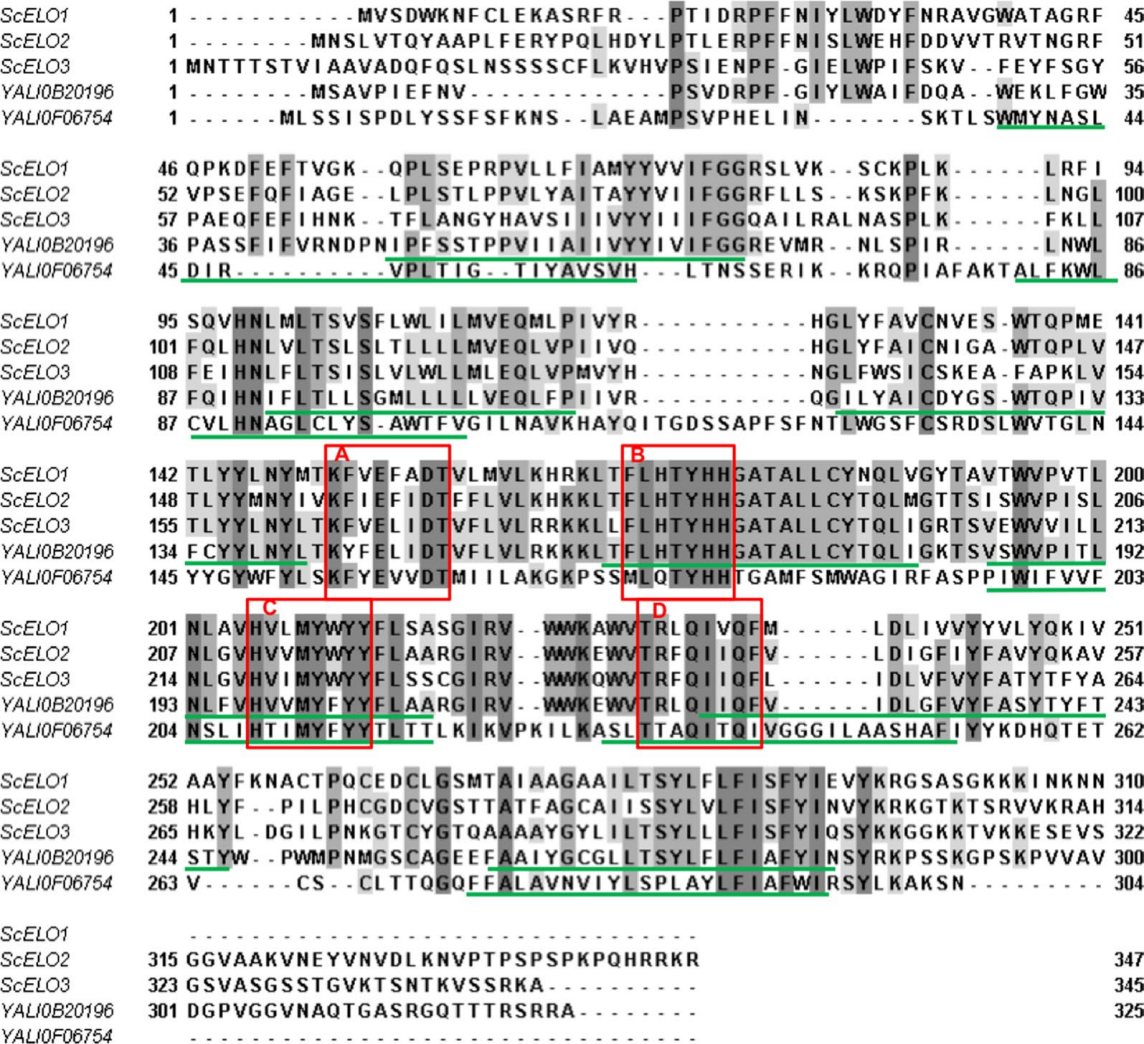
**A** Multiple sequence alignment of *Saccharomyces cerevisae* elongase*s* and *Yarrowia lipolytica* elongases. Accession numbers: ScELO1, P39540; ScELO2, P25358; ScELO3, P40319, YALI0B20196, Q6CDY7; YALI0F06754, Q6C2L8.The four motifs found in members of the ELO family are boxed (**A**–**D**) in red. Box **B** contains the histidine-rich region common to elongase enzymes. Transmembrane regions predicted using TMHMM Server v. 2.0 (http://www.cbs.dtu.dk/services/TMHMM/) are underlined (in green) for the elongase sequences of *Yarrowia lipolytica*

In *S. cerevisiae*, three ELO proteins were identified: ELO1 protein is involved in vivo, in the elongation of myristic acid (C14 FA) to palmitic acid (C16 FA) [32, 33] whereas ELO2 and ELO3 are involved in synthesis of VLCFA (C16/C18 to C26 FA), substrates for sphingolipids synthesis [24, 25]. On the contrary, ELO protein functions have not been described in *Y. lipolytica*. Our search for alignment with *Sc*ELO1, *Sc*ELO2 and *Sc*ELO3 protein sequences from *Saccharomyces cerevisiae* using tBLASTp for *Y. lipolytica* CLIB22 strain provided two proteins sequences: YALI0B20196p and YALI0F06754p, in agreement with the finding of Dujon et al. in 2004 [31]. YALI0B20196 and YALI0F06754 genes are located respectively on chromosome B and F and share only 25% identity. Alignment with *Sc*ELO1, *Sc*ELO2 and *Sc*ELO3 is presented in Fig. 1. Overall, YALI0B20196p share more than 50% identity with the three *Sc*ELO whereas YALI0F06754p less than 30% (Table 1). For YALI0F06754 we found higher identity with elongases involved in polyunsaturated fatty acids (PUFA): 32% with the C18∆9 PUFA elongase (ADD51571) of the microalgae *Isochrysis galbana* [34] and with the ∆5 elongase of the fungus *Thraustochytrium* aureum [35] (data not shown).

**Table 1:**
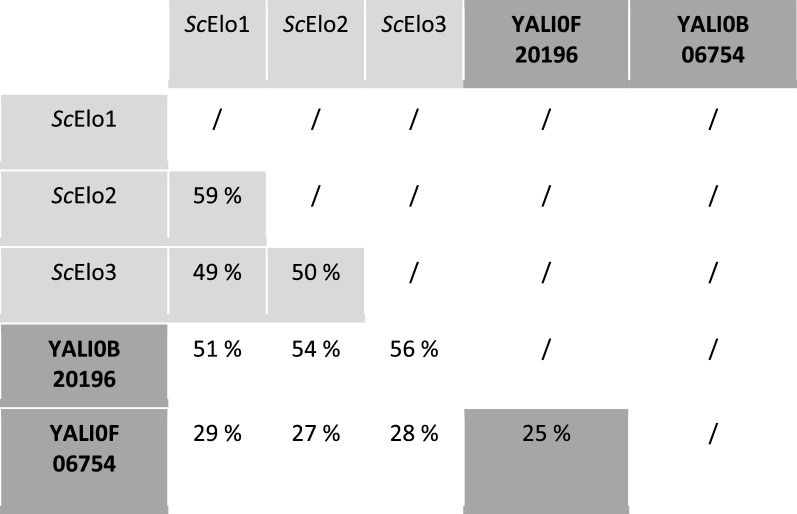
Percentage identity between elongases from *S. cerevisiae* and elongase from *Y. lipolytica*

Both *Yl*ELO sequences display conserved signature sequence motifs of the Elop family [28] as depicted in Fig. 1A. YALI0B20196p and YALI0F0654p display seven and five predicted transmembrane helix (TMH) regions and an histidine box motif: the canonical HXXHH and a variant QXXHH respectively, where XX are TY for both as observed in the three ScELO.

To avoid missing additional elongase sequences we performed tBlastn of putative fungal fatty acid elongases from *Aspergillus nidulans* (XP-658585), *Gibberella zeae* (XP_387072), and *Neurospora crassa* (XP_959004) or functionally characterized elongases from *Hansenula polymorpa* (AB194620), *Mortierella alpine* (AAF71789) [36] and *D. discoideum* (EU826171) [37]. None of these alignments allowed for the identification of another elongase protein in *Y. lipolytica*. As *Sc*ELO2 and *Sc*ELO3 have overlapping roles in *S. cerevisiae* [24], it seems thus reasonable to postulate that in *Yarrowia lipolytica* two ELO proteins can ensure the roles of the three *Sc*ELO enzymes in *S. cerevisiae*.

### Characterization of elongase function in *Y. lipolytica*

In order to elucidate the role of the two Elongases identified in *Y. lipolytica*, we constructed the strains ∆*pox* ∆YALI0F0654 and ∆*pox* ∆YALI0B20196. We found that the strain ∆*pox* ∆YALI0F0654 presents a normal growth whereas the ∆*pox* ∆YALI0B20196 strain growth presented an unusual phenotype, forming hyphae aggregated with branching and bulges (Additional file 1: Figure S1). This suggests an essential role of YALI0B20196 in *Y. lipolytica* as it was demonstrated for the simultaneous disruption of *Sc*ELO2 and *Sc*ELO3 in *S. cerevisiae* [38]. Taking into account these results, we can hypothesize for YALI0F0654 a similar role as *Sc*ELO1 in the elongation cycle of C14 FA into C16 FA in *Y. lipolytica*. Based on these results, we decided to name YALI0F0654 *Yl*ELO1 and YALI0B20196 *Yl*ELO2.

**Fig. 2:**
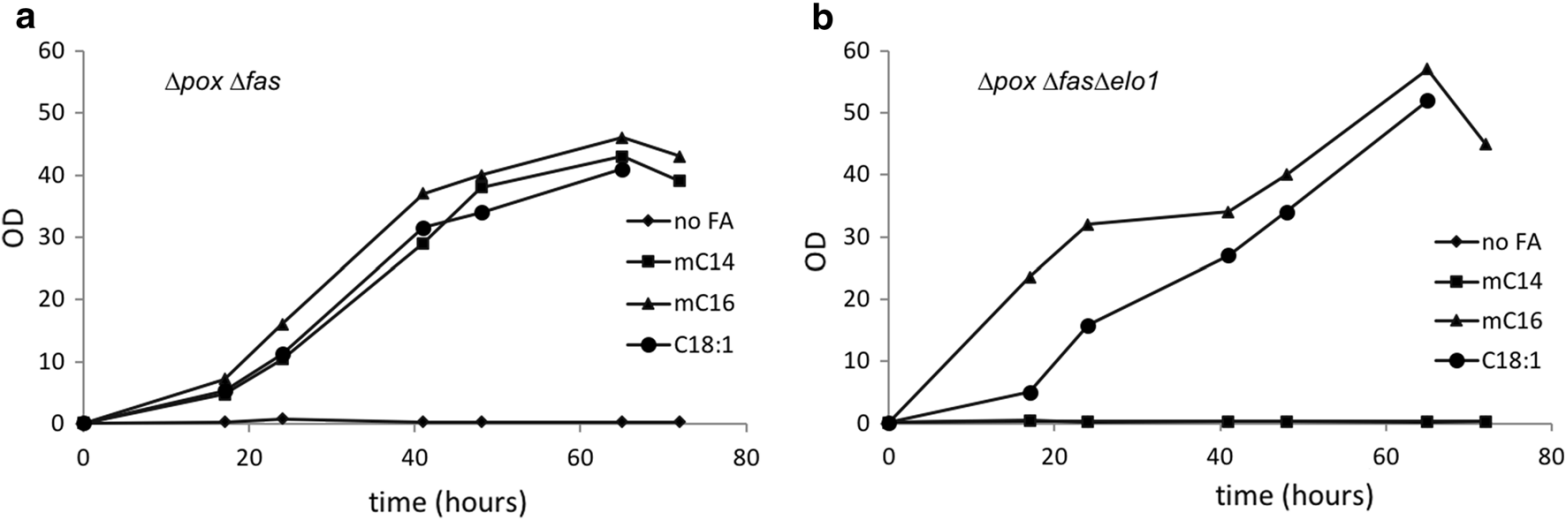
Growth of the strains ∆*pox* ∆*fas* (**a**) and ∆*pox* ∆*fas* ∆*elo1* (**b**) in rich medium YT2D5 (diamond) complemented with mC14:0 (square), mC16:0 (triangle) or C18:1 (circle)

**Fig. 3:**
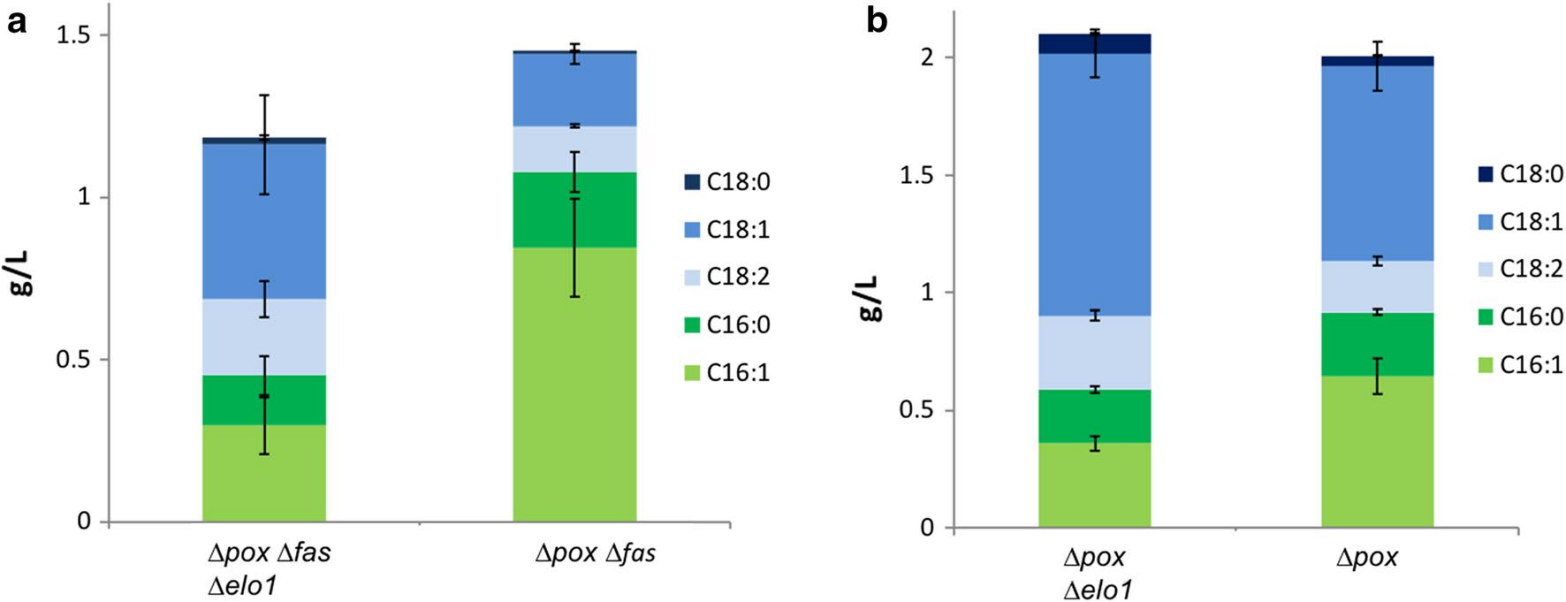
**a** Lipid profiles of the strains ∆*pox* ∆*fas* and ∆*pox* ∆*fas* ∆*elo1* grown in rich medium complemented with mC16:0 at 72 h. **b** Lipid profiles of the strains ∆*pox* and ∆*pox* ∆*elo1* grown in rich medium at 72 h. C16 fatty acids are depicted in green, C18 fatty acids in blue. Average and standard deviation are given for two clones cultivated separately

To verify *Yl*ELO1 function in *Y. lipolytica*, we investigated the growth of the ∆*pox* ∆*fas* strain (a strain that can neither synthesize nor degrade FAs) deleted or not for the *ELO1* gene, in rich YT2D5 medium supplemented with fatty acids methyl esters of various chain length sizes. Noteworthy, we use a ∆*pox* background in order to analyze FAs coming only from de novo synthesis. We found that the ∆*pox* ∆*fas* strain can grow on methyl myristate (mC14:0), methyl palmitate (mC16:0) and oleic acid (C18:1) (Fig. 2a) whereas the ∆*pox* ∆*fas* ∆*elo*1 strain cannot grow on mC14:0 but can grow on mC16:0 and C18:1 (Fig. 2b). This suggests that *Yl*ELO1 function is essential to elongate C14 FA into longer FAs to support the need for lipids essential to ensure growth of the cell and that *Yl*ELO1 is the only elongase capable of converting C14 into longer FA. The lipid profile of the strains ∆*pox* ∆*fas* ∆*elo1* and ∆*pox* ∆*fas* growing in the presence of mC16:0 were analyzed. We found that both strains presented similar growth and an equivalent total amount of lipid after 6 days (Fig. 3a). However, after 3 days, the ∆*pox* ∆*fas* ∆*elo1* had converted only 25.9 ± 1.9% of the exogenous C16 FAs into C18 FAs whereas 62.1 ± 0.9% had been converted by the strain ∆*pox* ∆*fas.* Because ∆*pox* ∆*fas* ∆*elo1* is capable of synthesizing C18 FAs from exogenous mC16, *Yl*ELO1 is likely not the only elongase in *Y. lipolytica* catalyzing the elongation of C16 FA to C18 FA (Fig. 3a).

In addition, we found that deletion of *Yl*ELO1 modifies the lipid profile of the strain, which is not the case when *Sc*ELO1 is deleted in *S. cerevisiae* [29]. Indeed, as depicted in Fig. 3b, C16 FAs species (C16:0 and C16:1) represent 27,9 ± 0.1% of total lipid content in the ∆*pox* strain and increase to 45,7 ± 0.4% of total lipid content in the ∆*pox* ∆*elo1* strain (both strains were cultivated on rich medium without FA complementation and were able to accumulate the same amount of total lipids). This suggests that *Yl*ELO1 may also be involved in the elongation of C16 FAs into C18 FAs. Taken together, these results give evidence for the first time that *Yl*ELO1 enzyme from *Y. lipolytica* is crucial in the elongation of C14 FA and C16 FA.

In order to confirm that ELO use saturated substrates, we analyzed the elongation products of the strains ∆*pox* ∆*fas* and ∆*pox* ∆*fas* ∆*elo1* grown with mC16:0 (Fig. 3a). Elongation of C16:0 will lead to C18:0, further desaturated into C18:1∆9, one of the main fatty acid produced by *Y. lipolytica* [39] whereas elongation of C16:1 will lead to C18:1∆11. Derivatization of the methyl ester FA products of both reactions (Fig. 3a) was performed to allow for localization of the position of the double bond and thus discrimination between C18:1∆9 and C18:1∆11. For both strains, GC–MS analysis yielded two major products at m/z 173 and 217 consistent with the predicted fragmentation properties of C18:1∆9 (Additional file 1: Figure S2). Only traces amount of C18:1∆11 was detected for both strains, strongly supporting the fact that *Yl*ELO uses saturated fatty acid as substrate.

### Analysis of MCFA production by FAS mutant of *Y. lipolytica*

**Fig. 4:**
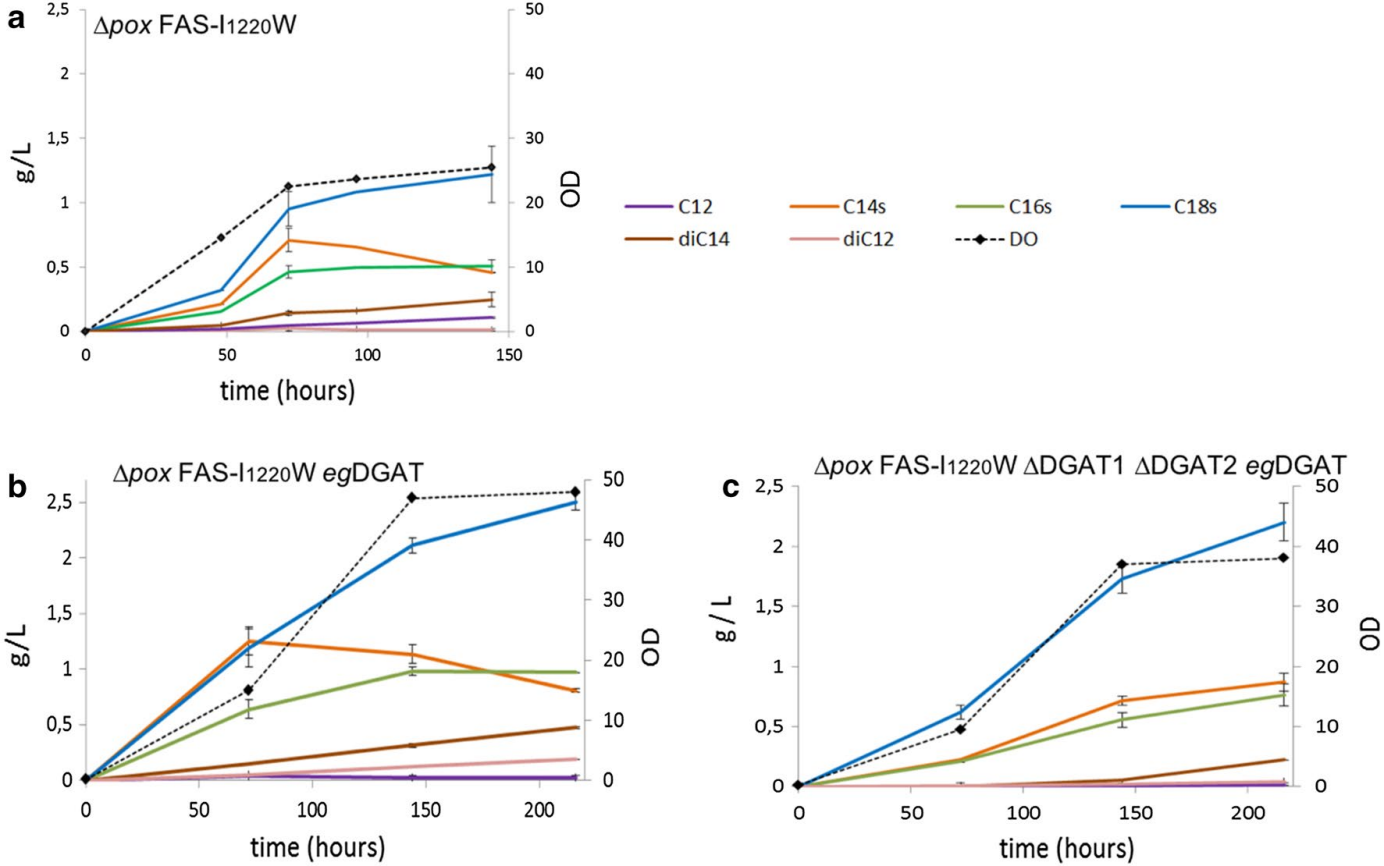
Kinetic of fatty acids synthesis by the strains ∆*pox* FAS-I_1220_W (**a**), ∆*pox* FAS-I_1220_W *eg*DGAT (**b**) and ∆*pox* FAS-I_1220_W ∆DGAT1 ∆DGAT2 *eg*DGAT (**c**). C18s corresponds to C18 fatty acid species (C18:0, C18:1 and C18:2) and is depicted in blue; C16s corresponds to C16 fatty acid species (C16:0 and C16:1) and is depicted in green. C14s corresponds to C14 fatty acid species (C14:0 and C14:1) and is depicted in orange. C12 FA is depicted in purple. diC12 is in brown and diC14 in pink. OD measure is given in a secondary vertical axis and is represented as black diamond markers on a dashed line. Average and standard deviation are given for two clones cultivated separately

We have previously generated FAS mutants capable of producing MCFA [19]. In particular, the strain ∆*pox* FAS-I_1220_W displays a mutation in the αFAS gene at position 1220, in the KS domain. We demonstrated that when the Isoleucine 1220 residue of the active site is replaced by a tryptophan, the length of the FAs synthesized is shifted toward shorter chains. In order to better understand the kinetic of MCFA synthesis by this strain, we cultivated ∆*pox* FAS-I_1220_W in Minimum Medium for optimal lipid accumulation and analyzed FA content along time. Results are depicted in Fig. 4a. ∆*pox* FAS-I_1220_W strain growth is slowed down compared with the growth of the ∆*pox* strain, reaching a maximum OD of 25 after 5 days versus an OD of 35 reached after 24 h for the ∆*pox* strain (data not shown). Looking into FAs content, we noticed that all FAs species increase during the first 3 days, following OD rise. When the growth reaches a plateau, we notice a decrease of C12 and C14 FAs whereas other FAs (C18s, C16s) continue to slowly accumulate (Fig. 4a). Interestingly for this strain, we found in the supernatant a significant amount of C12 and C14 dicarboxylic acids (DCA) (noted diC12 and diC14 respectively) and their concentrations kept increasing during the culture. After 3 days the growth stops and the FA changes we measure are likely coming from some FA pool rearrangement: the reduction of the amount of C14 FA (− 0.25 ± 0.09 g/L from day 3 to day 6) may be attributed to ω oxidation into diC14 (increase of 0.11 ± 0.05 g/L from day 3 to day 6) and its elongation into C16/C18 FAs by *Yl*ELO1 (increase of 0.27 ± 0.15 g/L from day 3 to day 6). Maximum C14 FAs content was measured after 3 days and reached 0.71 g/L ± 0.09 g/L and taken altogether, MCFA-DCA (C12 FA, C14 FAs and corresponding DCA) reached 0.92 ± 0.12 g/L, representing 39.4 ± 2% of total lipid content and 0.18 ± 0.04% g of MCFA-DCA/g cells.

### *Yl*ELO1 deletion in MCFA producers

We demonstrated that in *Y. lipolytica*, ELO1 enzyme is involved in the elongation cycle of C14 FA into C16 FA and of C16 FA into C18 FA. We decided to delete the gene in the strain ∆*pox* FAS-I_1220_W to potentially increase the amount of C14 FA produced. The mutation I_1220_W in the αFAS gene was performed in the ∆*pox* ∆*elo1* strain using TALEN technology as described by Rigouin et al. [19] and in the “Methods” section, giving the ∆*pox* ∆*elo1* αFAS-I_1220_W strain. Interestingly, fatty acid complementation was necessary to allow the growth of this strain, while complementation was not necessary for the previously characterized ∆*pox* FAS-I_1220_W strain. This suggests that ∆*pox* ∆*elo1* αFAS-I_1220_W has lost its ability to produce the fatty acids required for cell growth (C16 and C18 FAs). This would mean that the enzyme FAS-I_1220_W only synthesizes C14 FA and/or shorter FAs and that in a strain which does not express *Yl*ELO1, these shorter FAs cannot be elongated into C16 FA and C18 FA to support the growth. To verify this hypothesis, the strain was grown in YNB medium complemented with commercial oleic acid. Because FFAs may inhibit the synthesis of fatty acids [40] we decided to compare the lipid accumulation when adding 0.1% of oleic acid (OA) at the beginning of the culture or adding 0.02% daily. We found indeed that decreasing the amount of OA in the culture increases total lipid content (data not shown). However, even with only 0.02% of OA added daily, we could not reach more than 0.175 g MCFA-DCA g/L of culture after 6 days of growth with this strain (Additional file 1: Figure S3). In order to demonstrate that these MCFAs are the only products of de novo synthesis by FAS-I_1220_W, we cultivated the ∆*pox* ∆*elo1* αFAS-I_1220_W strain with _13_C-labelled glucose and 0.02% OA and followed lipid production in growing phase (after 3 days of culture). As expected, we found that 87% of de novo FA synthesized correspond to labeled C14 FA and 13% to labelled C16 FA (data not shown). Therefore, our hypothesis that FAS-I_1220_ W synthesizes mainly C14 is verified. Consequently, in the strain ∆*pox* FAS-I_1220_W expressing ELO1, the C16 and C18 FAs detected are certainly largely coming from C14 elongation by *Yl*ELO1.

As for ∆*pox* FAS-I_1220_W strain, the amount of C12 and C14 FAs decreases with time and the amount of the corresponding DCA secreted in the medium increases for the strain ∆*pox* ∆*elo1* FAS-I_1220_W. This suggests that C14 FA may not be efficiently accumulated as triacylglycerol (TAG) by the strain and rather undergo omega oxidation into DCA to promote their secretion in the medium.

### Increase MCFA accumulation with a diacylglycerol acyltransferase (DGAT) specific to MCFA

Medium chain acyl-CoAs accumulation as (TAG seems to be limited in *Y. lipolytica* since C14 FA content decreases even when its elongation is abolished. This implies that *Yarrowia lipolytica* DGAT specificity does not extend to medium chain acyl-CoAs. Consequently, another strategy was undertaken to promote MCFA accumulation as TAG by the αFAS mutant strains. Diglyceride acyltransferase (DGAT) are enzyme involved in the accumulation of acyl-Coa in TAG form [41] and recently a DGAT from *Elaeis guineensus* has been characterized and has shown preference for medium chain TAG accumulation in *Y. lipolytica* [42] when MCFA are supplied in the medium. We then constructed the strain ∆*pox* αFAS-I_1220_W *egDGAT* and followed MCFA accumulation over time. After 3 days of culture, C14 FA content reached 1.25 ± 0.12 g/L and total MCFA-DCA 1.50 ± 0.13 g/L (a 1.7 fold increase compared to ∆*pox* αFAS-I_1220_W strain) (Fig. 4b). C16s and C18s FAs species were also enhanced showing that *eg*DGAT promotes FA accumulation of Medium chain triacyl glycerol (MCTAG) but also of Long chain triacyl glycerol (LCTAG). Indeed, with this strain, the percentage of MCFA-DCA production represents 45 ± 0.01% of total lipid content and a 0.39 ± 0.01 g of MCFA-DCA/g cells was reached after 3 days of culture, a twofold increase in comparison to the ∆*pox* αFAS-I_1220_W strain.

Interestingly, when endogenous *Y. lipolytica* DGAT1 and DGAT2 are deleted and *eg*DGAT expressed in the strain ∆*pox* αFAS-I_1220_W (∆*pox* ∆*dgat1* ∆*dgat2* αFAS-I_1220_W *eg*DGAT), the lipid accumulation capacity (0.33 ± 0.04 g of total lipid/g of cells at day 6) is reduced with regard to the ∆*pox* αFAS-I_1220_W *egDGAT* (0.40 ± 0.01 g total lipid/g cells at day 6). However, the specific accumulation of C14 FA is enhanced compared to diC14 formation or elongation (Fig. 4c). Indeed, the ratio C14 FA/diC14 FA is 3.5 and 6.6 at the end of the growing phase (day 6) and 1.7 and 4 after 9 days for ∆*pox* αFAS-I_1220_W *eg*DGAT and ∆*pox* ∆*dgat1* ∆*dgat2* αFAS-I_1220_W *eg*DGAT respectively.

## Discussion

**Fig. 5:**
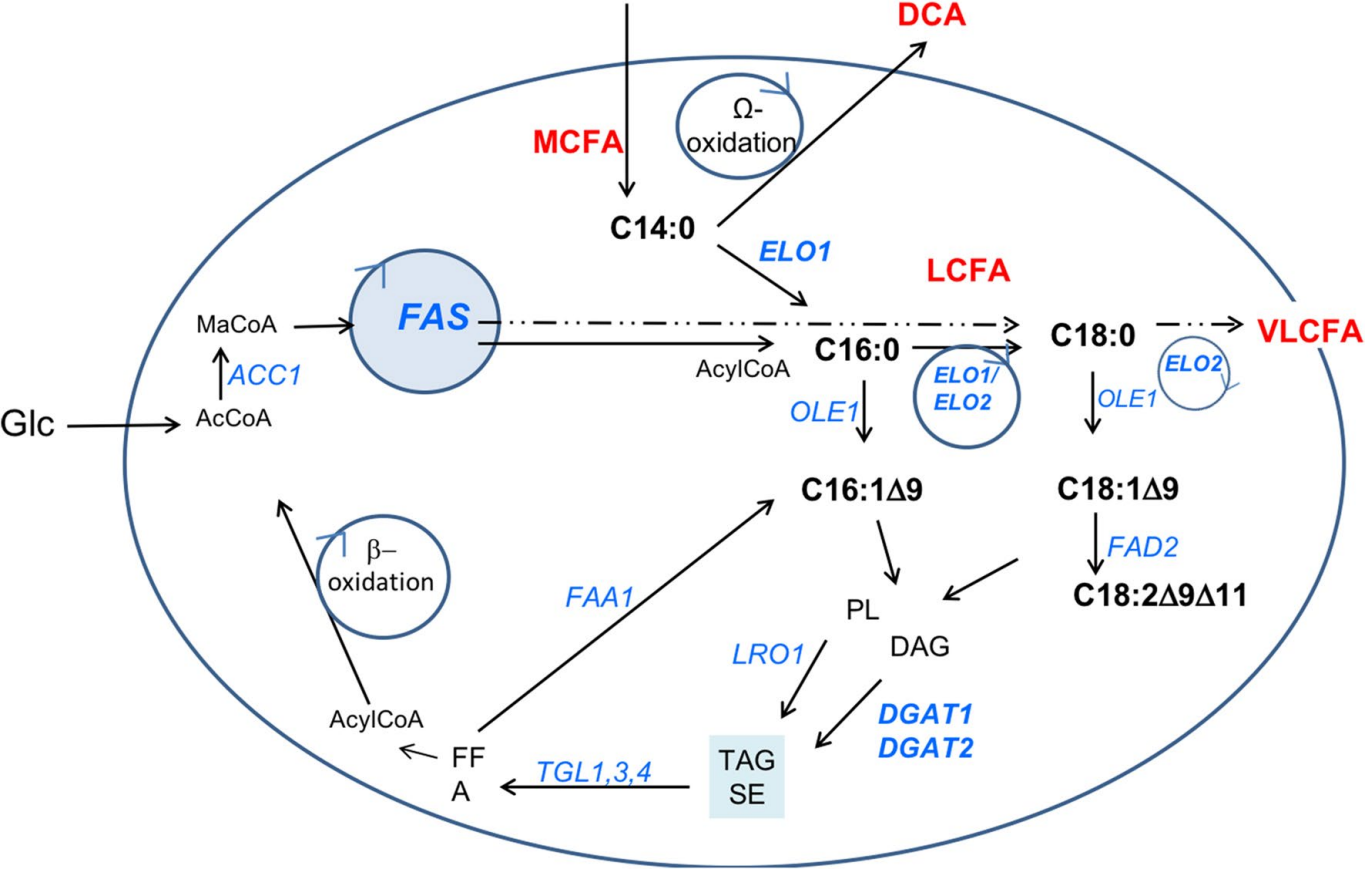
Lipid metabolism in *Y. lipolytica.* Schematic representation of the metabolic pathways of lipid synthesis, elongation, accumulation and degradation. Glc (glucose), AcCoa (acetyl-CoA), MaCoA (malonyl-CoA), FFA (free fatty acid), TAG (triacylglycerol), SE (steryl-esters), DAG (diacylglycerol), PL (phospholipids), MCFA (medium chain fatty acids), LCFA (long chain fatty acids), VLCFA (very long chain fatty acids). Gene names are in italic, in blue. ACC1 (acetyl-Coa carboxylase) FAS (fatty acid synthase), ELO (elongase), OLE1 (acyl-CoA desaturase), FAD2 (fatty acid desaturase), LRO (phospholipid DAG acyltransferase), TGL (triacylglycerol lipases), FAA1 (fatty acid-Coa synthetase), DGAT (DAG acyl transferase). Hypothetical routes discussed in this paper are depicted with a dashed arrow

In this paper, we describe for the first time the function of YALI0F06754 (that we named *Yl*Elo1) in *Yarrowia lipolytica*. We showed that *Yl*ELO1 is involved in the elongation cycle of C14 FA and C16 FA from exogenous and endogenous fatty acids. *S. cerevisiae* ScElo1 was described as being able to elongate in vitro both C14-CoA and C16-CoA. However, in vivo, only the elongation of C14 was confirmed. Indeed, the strain ∆*Scelo*1 showed the same growth rate and lipid profile as the wild type strain, suggesting that *Sc*ELO1 is not involved in the elongation of C16 FA [32]. In *Y. lipolytica*, we found that the ratio of C16/C18 was greatly enhanced when *Yl*ELO1 is deleted (Fig. 3b) and this difference cannot likely be explained by the ∆*pox* background of our strain. However, we showed evidence that another ELO enzyme may be also responsible for the elongation of C16 FA into C18 FA since the strain ∆*pox* ∆*fas* ∆*elo1* could synthesize C18 FA species when grown on mC16:0 (Fig. 3a). We found that *Yl*ELO2 deletion is highly detrimental for *Y. lipolytica*, as it is for concomitant deletion of *Sc*ELO2/*Sc*ELO3, the enzymes involved in VLCFA synthesis in *S. cerevisiae*. As we could not find evidence for the presence of a third ELO protein in *Y. lipolytica*, we hypothesize that YlELO2 (YALI0B20196) is likely the enzyme responsible for the elongation of C16 into C18 FA and for VLCFA synthesis (Fig. 5).

∆*pox* strain is naturally capable of synthesizing very small amounts C14 FA (< 0.01 g/L) [19] and it is interesting to notice that the amount of C14 FA synthesized by the ∆*pox* ∆*elo1* strain does not increase significantly. This allows us to conclude that FAS does not naturally produce MCFA. We showed that another elongase (likely YlELO2) synthesizes C18 from C16 (Fig. 3a) but cannot conclude whether FAS produces directly C18 FA or produces essentially C16 FA that are further elongated into C18 (Fig. 5). As ∆*pox* ∆*elo2* strain growth is compromised, in vitro rather that in vivo investigation should be carried on to unravel this mystery.

Noteworthy, Schneiter et al. [43] showed that ScELO1 can perform the elongation of exogenous C14:1∆9 to C16:1∆11 in *S. cerevisiae* proving its activity on unsaturated substrate. In our case, when ∆*pox* ∆*fas and* ∆*pox* ∆*elo1* ∆*fas* were grown on C16:0, only C18:1∆9 was detected. This suggests that even if C16:0 is efficiently desaturated into C16:1∆9 by both strains (Fig. 3a), only C16:0 is used as substrate by ELO enzymes (Fig. 5). In this context, C16:1 can be considered as an end product if one wants to focus on C18 FA species production. It would be of great interest to identify the two reductases and the dehydratase enzymes working downstream ELO enzyme to complete the elongation cycle (YALI0A06785p shares 58% identity with the potential 3-keto acyl-CoA reductase Ybr159wp from *S. cerevisiae* and YALI0A04983 shares 34% identity with the enoyl-CoA reductase Tsc13 from *S. cerevisiae*).

YlELO1 sequence is rather unusual compared to most ELO sequences. It shares the highest percentage of identity with hypothetical elongases protein of yeast species such as *Sugiyamaella* (53%), *Ascoidea rubescens* (48%) and *Saccharomycopsis fibuligera* (47%). Unlike most ELO proteins, YlElo1 has five predicted bilayer spanning sequences and the histidine box is located in hydrophilic areas and separated by hydrophobic domains (Fig. 1). In addition, the histidine box displays the motif QXXHH rather than the usual HXXHH found in most ELO proteins. The HXXHH motif is also found in enzymes that carry out oxidative reactions (e.g. fatty acid desaturases [44]). The front-end desaturase displays the motif variant QXXHH in which Q instead of H is an essential requirement for protein function [45]. In ELO proteins, the first H of the HXXHH motif does not seem to be important for elongase activity [29]. Interestingly, the variant motif QXXHH is found in the sequence of the C18-∆9-PUFA specific elongase from *Isochrysis galbana.* In this case, the glutamine residue replacing the first histidine of the canonical histidine box was mutated to either a histidine or an alanine and the author found that it triggered a decrease in activity but not an inactivation of the enzyme [34]. We also find this particular variant motif in ∆5-elongases from *Thraustochytrium* and *Schizochytrium*, elongases involved in PUFA synthesis [35]. Whether this position is important in YlElo1 activity has yet to be determined.

The results of this study underline the bottleneck for modification of fatty acid chain length in microorganisms. We show that the production of MCFAs via FAS engineering is successful but MCFA are synthesized at the expense of the LCFA essential for growth. It is then important to find a compromise between tailored FAs production and physiological requirement of biological membranes. In *Y. lipolytica*, to maximize MCFA production, one will need to differentiate FAS de novo synthesis to generate essential FAs and mutant FAS synthesis to generate the shorter chain FAs. With this approach, we could consider to compartmentalize MCFA production [46, 47] or trigger specific secretion of MCFA [18] to enhance productivity and MCFA yield.

Interestingly in our study, we found that part of the MCFAs produced undergo ω-oxidation. ω-oxidation has been described in *Y. lipolytica* [48] and acts as a rescue route in the peroxisome-deficient cell, generating oxygenated FA of higher solubility in water [49]. DCA can be synthesized in *Y. lipolytica* from hydrophobic substrate such n-alkane and fatty acids and are usually the products of bioconversion. Moreover, production of DCA from de novo synthesis has been recently described for the production of long chain dicarboxylic acid (LCDCA-16 and 18) using glycerol as the sole source of carbon [50] allowing the production of 0.3 g/L LCDCA in shake flask. In our study, we showed that the strain ∆pox FAS-I_1220_W *eg*DGAT can produce up to 0.47 ± 0.07 g/L MCDCA in shake flask which give to the strain a great potential and represents the first example to our knowledge of de novo synthesis of DCA with length from 10 to 14 carbons.

## Conclusion

Our study highlights the significance of elongase enzymes in the design of strains with modified lipid profile and the potential of *Y. lipolytica* to produce MCFA. We showed that depending on the targeted biotechnological application, MCFA fate can be driven by metabolic engineering toward accumulation or DCA production.

## Methods

### Media

Rich medium YPD (yeast extract 10 g/L, bactopeptone 10 g/L, glucose 10 g/L) was used for growing cells prior genomic DNA extraction and for growing precultures. Minimal medium YNB (glucose 10 g/L, YNB w/o AA 1.7 g/L, NH_4_Cl 5 g/L, phosphate buffer pH 6.8 50 mM, agar 15 g/L) was used to select colonies after transformation. The rich medium YT2D5 (yeast extract 10 g/L, bactotryptone 20 g/L, glucose 50 g/L) was used to grow cells for lipids content analysis.

The specific minimal medium for lipid accumulation was used to grow cells [glucose 80 g/L, ammonium sulfate 1.5 g/L, Phosphate buffer pH 6.8 100 mM, oligo elements: CoCl_2_ 0.5 mg/L, CuSO_4_ 0.9 mg/L, Na_2_MoO_4_ 0.06 mg/L, CaCl_2_ 23 mg/L, H_3_BO_3_ 3 mg/L, MnSO_4_ 3.8 mg/L, MgSO_4_ 10 mg/L, ZnSO_4_ 40 mg/L, FeSO_4_ 40 mg/L, vitamins (d-biotin 0.05 mg/L, pantothenic acid 1 mg/L, nicotinic acid 1 mg/L, Myo-inositol 25 mg/L, thiamine hydrochloride 1 mg/L, pyridoxol hydrochloride 1 mg/L, *p*-aminobenzoic acid 0.2 mg/L)].

### Fatty acid solution

Methyl myristate (mC14:0), methyl palmitate (mC16:0) and oleic acid (C18:1) (Fisherscientific A195-500) were emulsified by sonication in H2O and Tergitol at 20% (v/v).

### Strains constructions

The strain *Yarrowia lipolytica* JMY1233 (*MATa*, *leu2*-*270*, *ura3*-*302*, *xpr2*-*322*, Δ*pox*1-6), named ∆*pox* strain in this study was used. It has been deleted of the β-oxidation pathway [36] and is auxotroph for both uracil and leucine. This strain is used as a platform for the engineering of strains producing new or original lipids. β-Oxidation was removed to prevent degradation of any new types of fatty acid produced by the engineered strains. All cells are made competent and transformed using the Frozen-EZ Yeast Transformation II Kit™ (Zymoresearch).

### YALI0B20196 deletion (Elo2 deletion)

ELO2 disruption was carried out using CRISPR/Cas9 technology (unpublished data). Target designed was carried out with the online tool CRISPOR [51], which indicated the two sequences tgatcacaggtgcaaatcc and gtagaagtacatgacaacg (located on the ELO2 intron and exon respectively) as potential suitable target sites. The former sequence was incorporated in a generic pCg58 plasmid (i.e. a plasmid bearing a Cas9 gene under the control of a TEF promoter and a sgRNA gene under the control of an hybrid ScR1-tRNAgly promoter, with URA3 as a marker of selection) and the latter in a pg7 generic plasmid similar to the previous one, excepted with LEU2 as a marker of selection and without the Cas9 gene (Additional file 1: Method S1). The ∆*pox* strain was co-transformed with both plasmids and transformants selected on YNB plates. A few colonies were visible after 3 days of incubation at 28 °C and displayed normal growth, while the majority of colonies did not appear until 5 days and kept a very slow growth. Sequencing at the ELO2 locus of fast growing colonies indicated that the target site in the exon was identical to WT. Slow growing colonies were picked and grown on selective YNB plates for 9 days at 28 °C in order to generate enough biomass for PCR analysis. Sequencing at the locus indicated that the ELO2 gene of these transformants had been successfully disrupted as the sequence between the two target sites had been flipped (Additional file 1: Figure S4).

### YALI0F06754 deletion (*Yl*Elo1 deletion)

Gene deletion was performed on the ∆*pox* strain. Using a disruption cassette generated by PCR amplification as described by Fickers et al. [52]. The promoter (P) and terminator (T) regions were amplified from genomic DNA using primers listed in Additional file 1: Table S1 and fused by PCR with an I-*Sce*I restriction site at the junction. The PT resulting cassettes were then inserted into the PCR4RBlunt-TOPO vector (Life Technologies, Carlsbad, California) and the auxotrophic marker (LEU2) was then inserted into the vectors using the I-*Sce*I restriction sites to generate the corresponding PLT-ELO1 vector. The PLT-ELO1 disruption cassette (Additional file: Table S2) generated by *Not*I digestion of the plasmid was then introduced into the ∆*pox* strain. Transformants were selected on appropriate selective medium. Homologous recombination was confirmed by sequencing PCR products on ELO1 locus. Marker rescue was finally performed using the cre/loxP system after transformation with the replicative plasmid pUB4-CreI [52].

### FAS disruption

Disruption of the FAS gene was carried out using the TALEN plasmids pU18TAL_KSr and pL68TAL_KSl as described by Rigouin et al. [19] on both ∆*pox* and ∆*pox* ∆*elo1* strains giving rise to strains ∆*pox* ∆*fas* and ∆*pox* ∆*fas* ∆*elo1* strain respectively. Upon transformation, cells were selected on YNB medium complemented by 0.1% (v/v) oleic acid. Disruption was verified by PCR and confirmed by sequencing (GATC Biotech). The clones selected carried insertions or deletions leading to frameshift of the αFAS gene. The clones carrying the disrupted FAS are named ∆*fas* rather than FAS^−^ to facilitate reading.

### αFAS-I_1220_W mutation

The mutant αFAS-I_1220_W was constructed as described by Rigouin et al. [19] on the ∆*pox* ∆*elo1* strain using the TALEN plasmids pU18TAL_KSr and pL68TAL_KSl and the matrix I_1220_W to trigger homologous recombination, giving rise to strain ∆*pox* ∆*elo1* αFAS-I_1220_W. Homologous recombination was confirmed by sequencing of PCR products around the αFAS mutation.

### *eg*DGAT expressing strains

the plasmid JMP62 URA3 ex pTEF_egDGAT [42] was digested by *Not*I to generate the integration cassette. 500 ng of the digestion product were used to transform ∆*pox* FAS-I_1220_W and ∆*pox* ∆*elo1* FAS-I_1220_W. Transformations were plated on appropriate selective media. Random genome integration was verified by PCR using the primers Tef-F and Lip_R located inside the cassette (Additional file 1: Table S1).

### DGAT1 (YALI0E32769p) and DGAT2 (YALI0D07986p) deletions

Disruptions were carried out using CRISPR/Cas9 technology as described above for *Yl*ELO2 disruption. Potential suitable target sites for DGAT1 and DGAT2 genes were identified via CRISPOR and are listed in table S3 (Additional file 1: Table S3). DGAT1g1 sequence was incorporated in pCg58 plasmid and DGAT2 g2 into pg7. The ∆*pox* strain was co-transformed by both plasmids and transformants selected on YNB plates. The clone selected carried an eight bp deletion in each gene leading to frameshifts in both DGAT1 and DGAT2 genes (Additional file 1: Figure S5) and was named ∆DGAT1 ∆DGAT2 rather than DGAT1^−^ DGAT2^−^ to facilitate reading.

### Cultures, lipids extraction and analysis

All strains were made prototroph for further analysis of lipids content. They were grown in 20 mL of YT2D5 or 50 mL of minimum medium required for lipid accumulation at 28 °C for 8 days. 2 mL samples (cells + medium) were collected along time for growth measurement and were lyophilized for further analysis. When required, cells and medium were separated by centrifugation before analysis to discriminate between intracellular and extracellular fatty acids. Dry cells weight (DCW) was estimated from OD values. In our lab, we have determined that the ratio OD/DCW for a ∆*pox* strain is 4 ± 0.7. We applied this ratio on our OD values to evaluate the cells weight.

For each strain constructed, FA profiles were analyzed for two independent clones cultivated separately, after their extraction and transmethylation in hot acid methanol [53]. Briefly, 2 mL of a solution of methanol (with C17 FA prepared at 0.2 mg/mL as standard) with 2.5% sulfuric acid is added to the dried sample in addition to 1 mL of Toluene. Samples are heated at 80 °C for 3 h. Once the samples are cooled down, biphasic liquid extraction takes place using 1.5 mL of 0.5 M NaCl and 1.5 mL hexane (containing mC20 FA at 0.1 mg/mL as internal standard). Analyses are performed on organic phase with a gas chromatography coupled with Mass Spectrometry (GC–MS) *TRACE*™ 1310 equipped with the *TRACE*™ TR-5 column (Thermo-scientific). Standards of methyl fatty acids and methyl DCA were used to determine retention time and for quantification.

### Derivatization of fatty acid methyl esters

Standards C18:1∆11 and C18:1∆9 were prepared at 1 g/L in n-hexane. 50 µL of these samples were transferred in a 4 mL glass vial and 100 µL of dimethyl disulfide (DMDS) and 100 µL of a 6% solution of iodine in diethyl ether were added. The reaction was stirred at 37 °C for 40 h. 1 mL of a 5% solution of sodium thiosulfate and 1 mL of *n*-hexane were then added. The organic phase was isolated and completely dried under a stream of nitrogen before being resuspended into 100 µL of *n*-hexane and analyzed by GC–MS. Hexane fractions from transmethylation of the ∆*pox* ∆*fas* ∆*elo1* and ∆*pox* ∆*fas* strains reactions with mC16:0 after 3 days were used and were treated following the procedure described above.

## Additional file

**Additional file 1: Figure S1.** (A) Growth of the strains ∆*pox (square) and* ∆*pox*∆YALI0F06754 (∆elo1) (circle) in rich medium YT2D5. (B) phenotype of ∆*pox* (left side) and ∆*pox* ∆YALI0B20196 (∆*elo2*) (right side) strains observed on the microscope (40X objective lens). **Figure S2.** Characterization of the double bond position present in the product of ∆*Elo2* ∆*fas* strains grown in mC16 after 3 days of culture. Dimethyl disulfide derivatives of fatty acid methyl esters were prepared and analyzed as described in Materials and Methods. Mass spectrum of the peak corresponding to derivatized C18:1 methyl ester is depicted. **Figure S3.** kinetic of MCFA synthesis by the ∆*pox* ∆*elo1* FAS-I_1220_W strain complemented with 0.02% of Oleic acid every 24h. **Figure S4.** Sequencing data of the ∆*pox* ∆*elo1* (∆YALI0F06754) strain at targeted locus. Sequencing at the ELO2 locus was performed on two independent clones with the primers used for the PCR amplification of the ELO1 locus (ELO1_P and ELO1_T). The part highlighted in blue corresponds to the fragment that flipped between the two cut sites, in yellow are bases that are absent in one of the two disrupted clones. **Figure S5.** Sequencing data of the ∆*pox* ∆*dgat*1 ∆*dgat*2. DGAT1 sequencing was performed with the primers DGAT1-Verif-F and DGAT1-Verif-R. DGAT2 sequencing was performed with the primers DGAT2-Verif-F2 and DGAT2-Verif-R2. The wild type sequence of DGAT1 and DGAT2 is given and the parts highlighted in yellow correspond to the deletion in the ∆*pox* ∆*dgat*1 ∆*dgat*2 strain. **Table S1.** Primers and oligonucleotides used in this study. **Table S2.** Sequence of the PLT-ELO1 cassette designed to carry out ELO1 gene deletion. **Table S3.** Sequence of the guides used to carry out deletion of ELO2 gene or DGAT genes using CRISPR-Cas9 tool. **Method S1.** plasmids construction for CRISPR/Cas9 genome editing.

## Abbreviations

FAS: fatty acid synthase
FA: fatty acid
FFA: free fatty acid
MCFA: medium chain fatty acid
DCA: dicarboxylic acid
KS: ketoacyl synthase
VLCFA: very long chain fatty acid
ELO: elongase
FAA: free fatty acid
TAG: triacyl glycerol
DGAT: diglyceride acyltransferase
OD: optical density
TALEN: transcription activator-like effector nucleases
OA: oleic acid

## References

[1] Shields-Menard SA, Amirsadeghi M, French WT, Boopathy R (2018). A review on microbial lipids as a potential biofuel. Bioresour Technol.

[2] Sheng J, Feng X (2015). Metabolic engineering of yeast to produce fatty acid-derived biofuels: bottlenecks and solutions. Front Microbiol.

[3] Sarkar D, Shimizu K (2015). An overview on biofuel and biochemical production by photosynthetic microorganisms with understanding of the metabolism and by metabolic engineering together with efficient cultivation and downstream processing. Bioresour Bioprocess.

[4] Majidian P, Tabatabaei M, Zeinolabedini M, Naghshbandi MP, Chisti Y (2018). Metabolic engineering of microorganisms for biofuel production. Renew Sustain Energy Rev.

[5] Adrio JL (2017). Oleaginous yeasts: promising platforms for the production of oleochemicals and biofuels. Biotechnol Bioeng.

[6] Shi S, Zhao H. Metabolic engineering of oleaginous yeasts for production of fuels and chemicals. Front Microbiol. 2017;8. https://www.ncbi.nlm.nih.gov/pmc/articles/PMC5682390/. Accessed 10 Apr 2018 10.3389/fmicb.2017.02185 PMC5682390 29167664

[7] Madzak C (2015). *Yarrowia lipolytica*: recent achievements in heterologous protein expression and pathway engineering. Appl Microbiol Biotechnol.

[8] Zhu Z, Zhou YJ, Krivoruchko A, Grininger M, Zhao ZK, Nielsen J (2017). Expanding the product portfolio of fungal type I fatty acid synthases. Nat Chem Biol.

[9] Ledesma-Amaro R, Nicaud J-M (2016). *Yarrowia lipolytica* as a biotechnological chassis to produce usual and unusual fatty acids. Prog Lipid Res.

[10] Schwartz CM, Hussain MS, Blenner M, Wheeldon I (2016). Synthetic RNA polymerase III promoters facilitate high-efficiency CRISPR-Cas9-mediated genome editing in *Yarrowia lipolytica*. ACS Synth Biol.

[11] Gao S, Han L, Zhu L, Ge M, Yang S, Jiang Y (2014). One-step integration of multiple genes into the oleaginous yeast *Yarrowia lipolytica*. Biotechnol Lett.

[12] Qiao K, Wasylenko TM, Zhou K, Xu P, Stephanopoulos G (2017). Lipid production in *Yarrowia lipolytica* is maximized by engineering cytosolic redox metabolism. Nat Biotechnol.

[13] Friedlander J, Tsakraklides V, Kamineni A, Greenhagen EH, Consiglio AL, MacEwen K (2016). Engineering of a high lipid producing *Yarrowia lipolytica* strain. Biotechnol Biofuels.

[14] Blazeck J, Hill A, Liu L, Knight R, Miller J, Pan A (2014). Harnessing *Yarrowia lipolytica* lipogenesis to create a platform for lipid and biofuel production. Nat Commun.

[15] Xie D, Jackson EN, Zhu Q (2015). Sustainable source of omega-3 eicosapentaenoic acid from metabolically engineered *Yarrowia lipolytica*: from fundamental research to commercial production. Appl Microbiol Biotechnol.

[16] Xu P, Qiao K, Ahn WS, Stephanopoulos G (2016). Engineering *Yarrowia lipolytica* as a platform for synthesis of drop-in transportation fuels and oleochemicals. Proc Natl Acad Sci.

[17] Wang W, Wei H, Knoshaug E, Van Wychen S, Xu Q, Himmel ME (2016). Fatty alcohol production in *Lipomyces starkeyi* and *Yarrowia lipolytica*. Biotechnol Biofuels.

[18] Ledesma-Amaro R, Dulermo R, Niehus X, Nicaud J-M (2016). Combining metabolic engineering and process optimization to improve production and secretion of fatty acids. Metab Eng.

[19] Rigouin C, Gueroult M, Croux C, Dubois G, Borsenberger V, Barbe S (2017). Production of medium chain fatty acids by *Yarrowia lipolytica*: combining molecular design and TALEN to engineer the fatty acid synthase. ACS Synth Biol.

[20] Rutter CD, Zhang S, Rao CV (2015). Engineering *Yarrowia lipolytica* for production of medium-chain fatty acids. Appl Microbiol Biotechnol.

[21] Allouche Y, Cameleyre X, Guillouet S, Hulin S, Thevenieau F, Akomia L (2013). ProBio3 project: how to achieve scientific and technological challenges to boost the sustainable microbial production of lipids as biojet fuel and chemical compounds. OCL.

[22] Leibundgut M, Maier T, Jenni S, Ban N (2008). The multienzyme architecture of eukaryotic fatty acid synthases. Curr Opin Struct Biol.

[23] Tehlivets O, Scheuringer K, Kohlwein SD (2007). Fatty acid synthesis and elongation in yeast. Biochim Biophys Acta.

[24] Denic V, Weissman JS (2007). A molecular caliper mechanism for determining very long-chain fatty acid length. Cell.

[25] Oh CS, Toke DA, Mandala S, Martin CE (1997). ELO2 and ELO3, homologues of the *Saccharomyces cerevisiae* ELO1 gene, function in fatty acid elongation and are required for sphingolipid formation. J Biol Chem.

[26] Schneiter R, Brügger B, Amann CM, Prestwich GD, Epand RF, Zellnig G (2004). Identification and biophysical characterization of a very-long-chain-fatty-acid-substituted phosphatidylinositol in yeast subcellular membranes. Biochem J.

[27] Heath RJ, Rock CO (2002). The Claisen condensation in biology. Nat Prod Rep.

[28] Jakobsson A, Westerberg R, Jacobsson A (2006). Fatty acid elongases in mammals: their regulation and roles in metabolism. Prog Lipid Res.

[29] Hernandez-Buquer S, Blacklock BJ (2013). Site-directed mutagenesis of a fatty acid elongase ELO-like condensing enzyme. FEBS Lett.

[30] Gajewski J, Pavlovic R, Fischer M, Boles E, Grininger M (2017). Engineering fungal de novo fatty acid synthesis for short chain fatty acid production. Nat Commun.

[31] Dujon B, Sherman D, Fischer G, Durrens P, Casaregola S, Lafontaine I (2004). Genome evolution in yeasts. Nature.

[32] Toke DA, Martin CE (1996). Isolation and characterization of a gene affecting fatty acid elongation in *Saccharomyces cerevisiae*. J Biol Chem.

[33] Dittrich F, Zajonc D, Hühne K, Hoja U, Ekici A, Greiner E (1998). Fatty acid elongation in yeast—biochemical characteristics of the enzyme system and isolation of elongation-defective mutants. Eur J Biochem.

[34] Qi B, Fraser TCM, Bleakley CL, Shaw EM, Stobart AK, Lazarus CM (2003). The variant “his-box” of the C18-Delta9-PUFA-specific elongase IgASE1 from Isochrysis galbana is essential for optimum enzyme activity. FEBS Lett.

[35] Nagano N, Sakaguchi K, Taoka Y, Okita Y, Honda D, Ito M (2011). Detection of genes involved in fatty acid elongation and desaturation in thraustochytrid marine eukaryotes. J Oleo Sci.

[36] Sakuradani E, Nojiri M, Suzuki H, Shimizu S (2009). Identification of a novel fatty acid elongase with a wide substrate specificity from arachidonic acid-producing fungus *Mortierella alpina* 1S-4. Appl Microbiol Biotechnol.

[37] Blacklock BJ, Kelley D, Patel S (2008). A fatty acid elongase ELO with novel activity from *Dictyostelium discoideum*. Biochem Biophys Res Commun.

[38] Silve S, Leplatois P, Josse A, Dupuy PH, Lanau C, Kaghad M (1996). The immunosuppressant SR 31747 blocks cell proliferation by inhibiting a steroid isomerase in *Saccharomyces cerevisiae*. Mol Cell Biol.

[39] Beopoulos A, Cescut J, Haddouche R, Uribelarrea J-L, Molina-Jouve C, Nicaud J-M (2009). *Yarrowia lipolytica* as a model for bio-oil production. Prog Lipid Res.

[40] Ogiwara H, Tanabe T, Nikawa J, Numa S (1978). Inhibition of rat-liver acetyl-coenzyme-A carboxylase by palmitoyl-coenzyme A. Formation of equimolar enzyme-inhibitor complex. Eur J Biochem.

[41] Beopoulos A, Haddouche R, Kabran P, Dulermo T, Chardot T, Nicaud J-M (2012). Identification and characterization of DGA2, an acyltransferase of the DGAT1 acyl-CoA:diacylglycerol acyltransferase family in the oleaginous yeast *Yarrowia lipolytica*. New insights into the storage lipid metabolism of oleaginous yeasts. Appl Microbiol Biotechnol.

[42] Aymé L, Jolivet P, Nicaud J-M, Chardot T (2015). Molecular characterization of the *Elaeis guineensis* medium-chain fatty acid diacylglycerol acyltransferase DGAT1-1 by heterologous expression in *Yarrowia lipolytica*. PLoS ONE.

[43] Schneiter R, Tatzer V, Gogg G, Leitner E, Kohlwein SD (2000). Elo1p-dependent carboxy-terminal elongation of C14:1Delta(9) to C16:1Delta(11) fatty acids in *Saccharomyces cerevisiae*. J Bacteriol.

[44] Shanklin J, Cahoon EB (1998). Desaturation and related modifications of fatty acids. Annu Rev Plant Physiol Plant Mol Biol.

[45] Sayanova O, Beaudoin F, Libisch B, Castel A, Shewry PR, Napier JA (2001). Mutagenesis and heterologous expression in yeast of a plant Delta6-fatty acid desaturase. J Exp Bot.

[46] Lau YH, Giessen TW, Altenburg WJ, Silver PA (2018). Prokaryotic nanocompartments form synthetic organelles in a eukaryote. Nat Commun.

[47] Avalos JL, Fink GR, Stephanopoulos G (2013). Compartmentalization of metabolic pathways in yeast mitochondria improves the production of branched-chain alcohols. Nat Biotechnol.

[48] Gatter M, Förster A, Bär K, Winter M, Otto C, Petzsch P (2014). A newly identified fatty alcohol oxidase gene is mainly responsible for the oxidation of long-chain ω-hydroxy fatty acids in *Yarrowia lipolytica*. FEMS Yeast Res.

[49] Endoh-Yamagami S, Hirakawa K, Morioka D, Fukuda R, Ohta A (2007). Basic helix-loop-helix transcription factor heterocomplex of Yas1p and Yas2p regulates cytochrome P450 expression in response to alkanes in the yeast *Yarrowia lipolytica*. Eukaryot Cell.

[50] Abghari A, Madzak C, Chen S (2017). Combinatorial engineering of *Yarrowia lipolytica* as a promising cell biorefinery platform for the de novo production of multi-purpose long chain dicarboxylic acids. Fermentation.

[51] Haeussler M, Schönig K, Eckert H, Eschstruth A, Mianné J, Renaud J-B (2016). Evaluation of off-target and on-target scoring algorithms and integration into the guide RNA selection tool CRISPOR. Genome Biol.

[52] Fickers P, Le Dall MT, Gaillardin C, Thonart P, Nicaud JM (2003). New disruption cassettes for rapid gene disruption and marker rescue in the yeast *Yarrowia lipolytica*. J Microbiol Methods.

[53] Browse J, Kunst L, Anderson S, Hugly S, Somerville C (1989). A mutant of Arabidopsis deficient in the chloroplast 16:1/18:1 desaturase. Plant Physiol.

