# Supplementary material for: Increasing medium chain fatty acids production in Yarrowia lipolytica by metabolic engineering
Source: Microb Cell Fact. 2018 Sep 10;17:142. doi: 10.1186/s12934-018-0989-5 (PMC6130074; doi:10.1186/s12934-018-0989-5)
Supplement: Supplementary file 1 — Additional file 1: Figure S1. (A) Growth of the strains ∆pox (square) and ∆pox∆YALI0F06754 (∆elo1) (circle) in rich medium YT2D5. (B) phenotype of ∆pox (left side) and ∆pox ∆YALI0B20196 (∆elo2) (right side) strains observed on the microscope (40X objective lens). Figure S2. Characterization of the double bond position present in the product of ∆Elo2 ∆fas strains grown in mC16 after 3 days of culture. Dimethyl disulfide derivatives of fatty acid methyl esters were prepared and analyzed as described in Materials and Methods. Mass spectrum of the peak corresponding to derivatized C18:1 methyl ester is depicted. Figure S3. kinetic of MCFA synthesis by the ∆pox ∆elo1 FAS-I1220W strain complemented with 0.02% of Oleic acid every 24h. Figure S4. Sequencing data of the ∆pox ∆elo1 (∆YALI0F06754) strain at targeted locus. Sequencing at the ELO2 locus was performed on two independent clones with the primers used for the PCR amplification of the ELO1 locus (ELO1_P and ELO1_T). The part highlighted in blue corresponds to the fragment that flipped between the two cut sites, in yellow are bases that are absent in one of the two disrupted clones. Figure S5. Sequencing data of the ∆pox ∆dgat1 ∆dgat2. DGAT1 sequencing was performed with the primers DGAT1-Verif-F and DGAT1-Verif-R. DGAT2 sequencing was performed with the primers DGAT2-Verif-F2 and DGAT2-Verif-R2. The wild type sequence of DGAT1 and DGAT2 is given and the parts highlighted in yellow correspond to the deletion in the ∆pox ∆dgat1 ∆dgat2 strain. Table S1. Primers and oligonucleotides used in this study. Table S2. Sequence of the PLT-ELO1 cassette designed to carry out ELO1 gene deletion. Table S3. Sequence of the guides used to carry out deletion of ELO2 gene or DGAT genes using CRISPR-Cas9 tool. Method S1. plasmids construction for CRISPR/Cas9 genome editing. [file 12934_2018_989_MOESM1_ESM.docx]

**Additional file 1**

**A**

**
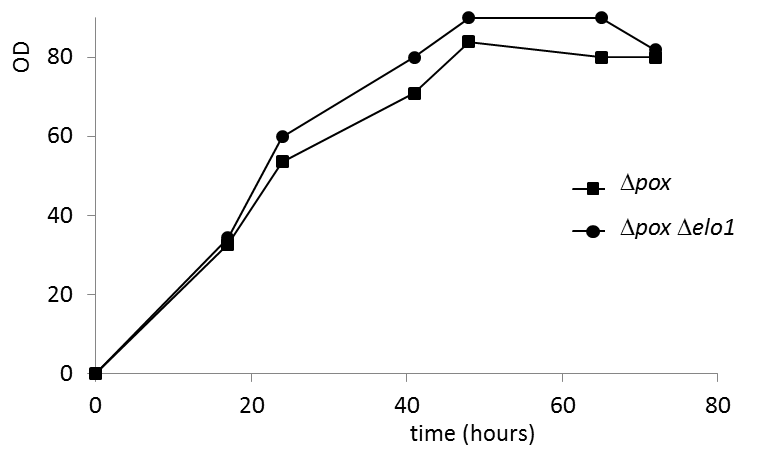
**

**B**


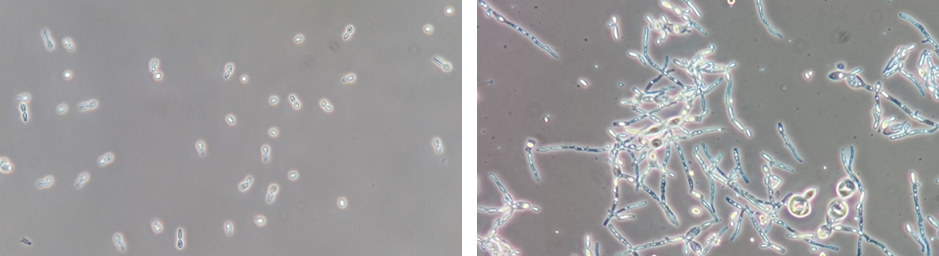


**Figure S1**: (A) Growth of the strains Δ*pox (square) and* Δ*pox*ΔYALI0F06754 (Δelo1) (circle) in rich medium YT2D5. (B) phenotype of Δ*pox* (A) and Δ*pox* ΔYALI0B20196 (Δ*elo2*) (B) strains observed on the microscope (40X objective lens)


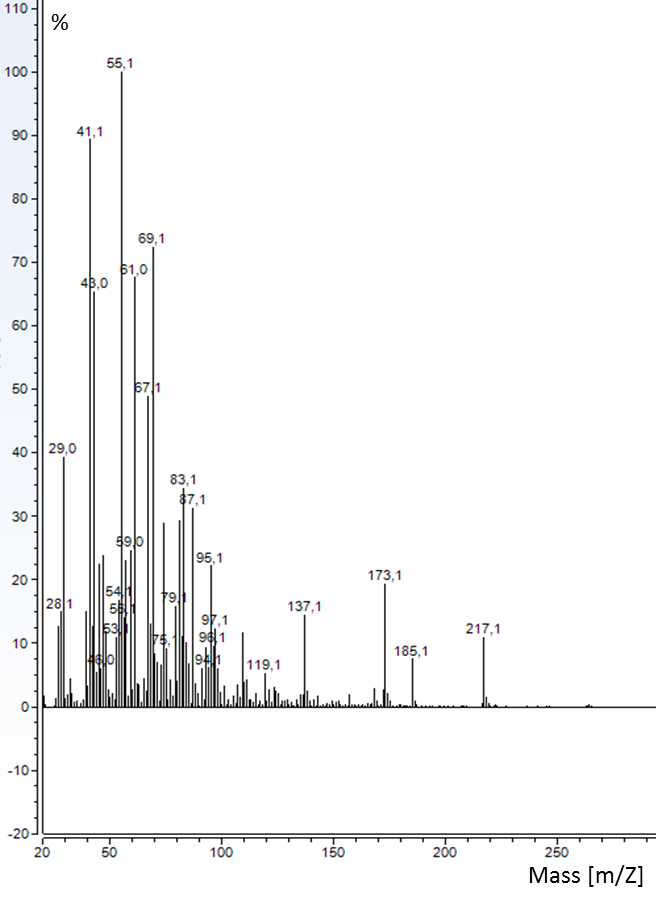


**Figure S2**: Characterization of the double bond position present in the product of Δ*Elo2* Δ*fas* strains grown in mC16 after 3 days of culture. Dimethyl disulfide derivatives of fatty acid methyl esters were prepared and analyzed as described in Materials and Methods. Mass spectrum of the peak corresponding to derivatized C18:1 methyl ester is depicted.


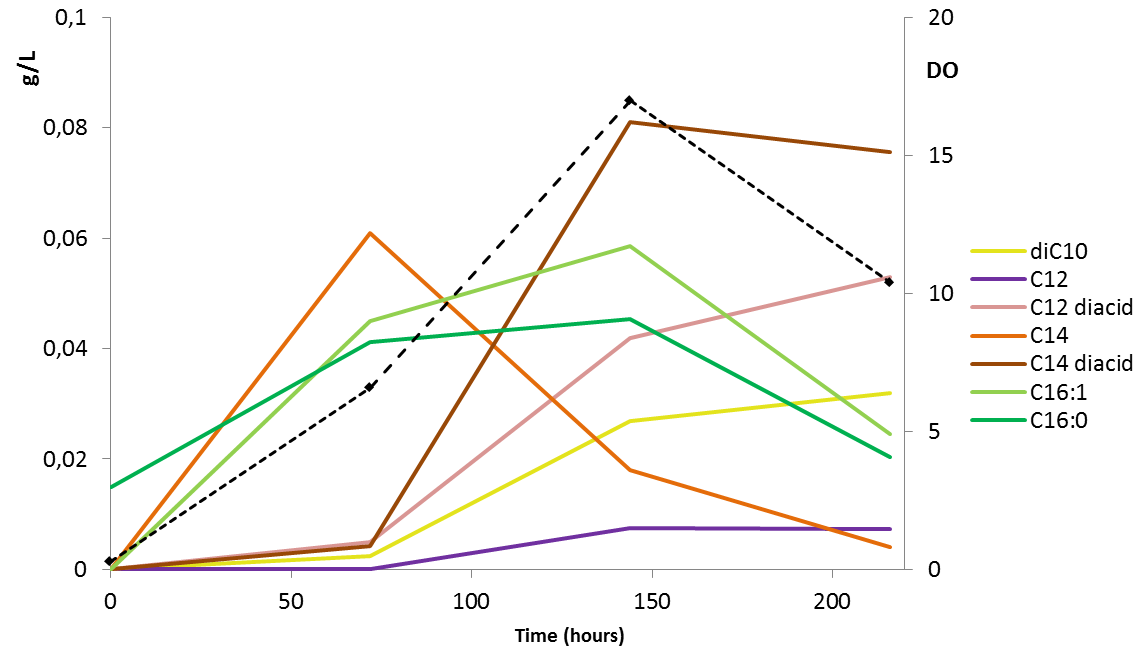


**Figure S3**: kinetic of MCFA synthesis by the Δ*pox* Δ*elo1* FAS-I_1220_W strain complemented with 0.02% of Oleic acid every 24h.

ttcacacgctctttttttccatgacctgcgtgccctaacctcggagaagcccaaaaccggcataacgcaaaagtctctttcgttttccgcacaagcaaaacctcctttcgcttctcgccagttgtactctcgttgcactgaaaacacgtttgcttggcgacagaaaaatgagtgagtatttcccacaaaacaaatgcccccagcagcgaacacgccccgacaccgatacacgcagcagcgcggaggtgacacaaatgcctcggaacgtggacaaacaggttaagggtgatggggacccaagagaccgaggtcttaccaatgagctgggtgtagcacagaagagcagtggcaccatggtggtaggtgtggaggaaagtcagcttcttctttcgcagcacaaggaaaacggtgtcgatcagctcaaagtacttggtcaggtagttgaggtagtagcagaagacaatgggctgagtccaagatccgtagtcgcagatggcgtagaggataccctgtcggacaatgatggggaagagctgctcaacgaggaggaggaggagcataccggacagaagggtgaggaagatgttgtggatctggaagagccagttgagtcggatgggagacaggtttcgcatcacctctcggccgccaaagatgacaatgtagtacacaatgatggcaatgatcacgggaggggtagaggaaaaggggatgttggggtcatttcgcacgaaaatgaaagaggacgcgggccagccgaaaagcttctcccaggcctggtcaaagatggcccagaggtagataccaaagggtcggtccacggaggggacgttgaattcaatagggacggcgcctgagttagtatagagcggatagtaaaggtgtgacagaggtcgtgtcccaaaaaggacgtcacgtcagtgttgtggcggccaccacatagtagtgacacgaggcttgttgtcacgtatatgttcggggtttgcctcacttcgccttgtatggattttgtcaaacatgagatcggaagtaacgatatggatcatgcgacatgtgagagacgtagatagatcaaggggcgaggtaggatttgtattatccatccgttactgtctctgcgtattcgtttctgttgtccggcgattaaaccccaaatctgccacgttttgttgtacgcgacgcgtagatcaggggtcaaatcaccccgcgacgacgataacagcgcttgagctgagtgttggtgacacaactaccggtagtatccccggtcgatcatcttctggctcgaggtgtcctcctaaaacatctgtttcgttgactgaatggtcgattacacaggacgtgtgtcccatgtcgacgtaacgtgttcatgatcacaggtgcaaattgtcatgtacttctactacttcctggctgcgcgaggtatccgagtgtggtggaaggagtgggtcacccggctccagatcatccagttcgttatcgatcttggatttgtctactttgcctcttacacctacttcacctctacctactggccctggatgcccaacatgggctcttgtgccggcgaggagtttgctgctatttacggctgtggtctgctgacctcttacctcttcctcttcatcgccttctacatcaactcttaccgaaagccctcttccaagggaccttccaagcctgttgttgctgtcgatggccctgttggcggcgtcaacgcccagactggtgcttctcgaggccagaccactacccgatctcgacgagcataaagaggggtatgaacagtgatacaaaaaagcagtttttactgtagtagattagataataaatgctctttcgaacagtcagccattcggccgccgagtggctcacgtagaatgccatatgtgatcttcttaaccatattgtataat

**Figure S4**: Sequencing data of the Δ*pox* Δ*elo1* (ΔYALI0F06754) strain at targeted locus. Sequencing at the ELO2 locus was performed on two independent clones with the primers used for the PCR amplification of the ELO1 locus (ELO1_P and ELO1_T). The part highlighted in blue corresponds to the fragment that flipped between the two cut sites, in yellow are bases that are absent in one of the two disrupted clones

>WT_DGAT1

atgactatcgactcacaatactacaagtcgcgagacaaaaacgacacggcacccaaaatcgcgggaatccgatatgccccgctatcgacaccattactcaaccgatgtgagaccttctctctggtctggcacattttcagcattcccactttcctcacaattttcatgctatgctgcgcaattccactgctctggccatttgtgattgcgtatgtagtgtacgctgttaaagacgactccccgtccaacggaggagtggtcaagcgatactcgcctatttcaagaaacttcttcatctggaagctctttggccgctacttccccataactctgcacaagacggtggatctggagcccacgcacacatactaccctctggacgtccaggagtatcacctgattgctgagagatactggccgcagaacaagtacctccgagcaatcatctccaccatcgagtactttctgcccgccttcatgaaacggtctctttctatcaacgagcaggagcagcctgccgagcgagatcctctcctgtctcccgtttctcccagctctccgggttctcaacctgacaagtggattaaccacgacagcagatatagccgtggagaatcatctggctccaacggccacgcctcgggctccgaacttaacggcaacggcaacaatggcaccactaaccgacgacctttgtcgtccgcctctgctggctccactgcatctgattccacgcttcttaacgggtccctcaactcctacgccaaccagatcattggcgaaaacgacccacagctgtcgcccacaaaactcaagcccactggcagaaaatacatcttcggctaccacccccacggcattatcggcatgggagcctttggtggaattgccaccgagggagctggatggtccaagctctttccgggcatccctgtttctcttatgactctcaccaacaacttccgagtgcctctctacagagagtacctcatgagtctgggagtcgcttctgtctccaagaagtcctgcaaggccctcctcaagcgaaaccagtctatctgcattgtcgttggtggagcacaggaaagtcttctggccagacccggtgtcatggacctggtgctactcaagcgaaagggttttgttcgacttggtatggaggtcggaaatgtcgcccttgttcccatcatggcctttggtgagaacgacctctatgaccaggttagcaacgacaagtcgtccaagctgtaccgattccagcagtttgtcaagaacttccttggattcacccttcctttgatgcatgcccgaggcgtcttcaactacgatgtcggtcttgtcccctacaggcgacccgtcaacattgtggttggttcccccattgacttgccttatctcccacaccccaccgacgaagaagtgtccgaataccacgaccgatacatcgccgagctgcagcgaatctacaacgagcacaaggatgaatatttcatcgattggaccgaggagggcaaaggagccccagagttccgaatgattgagtaa

>WT_DGAT2

atggaagtccgacgacgaaaaatcgacgtgctcaaggcccagaaaaacggctacgaatcgggcccaccatctcgacaatcgtcgcagccctcctcaagagcatcgtccagaacccgcaacaaacactcctcgtccaccctgtcgctcagcggactgaccatgaaagtccagaagaaacctgcgggacccccggcgaactccaaaacgccattcctacacatcaagcccgtgcacacgtgctgctccacatcaatgctttcgcgcgattatgacggctccaaccccagcttcaagggcttcaaaaacatcggcatgatcattctcattgtgggaaatctacggctcgcattcgaaaactacctcaaatacggcatttccaacccgttcttcgaccccaaaattactccttccgagtggcagctctcaggcttgctcatagtcgtggcctacgcacatatcctcatggcctacgctattgagagcgctgccaagctgctgttcctctctagcaaacaccactacatggccgtggggcttctgcataccatgaacactttgtcgtccatctcgttgctgtcctacgtcgtctactactacctgcccaaccccgtggcaggcacaatagtcgagtttgtggccgttattctgtctctcaaactcgcctcatacgccctcactaactcggatctccgaaaagccgcaattcatgcccagaagctcgacaagacgcaagacgataacgaaaaggaatccacctcgtcttcctcttcttcagatgacgcagagactttggcagacattgacgtcattcctgcatactacgcacagctgccctacccccagaatgtgacgctgtcgaacctgctgtacttctggtttgctcccacactggtctaccagcccgtgtaccccaagacggagcgtattcgacccaagcacgtgatccgaaacctgtttgagctcgtctctctgtgcatgcttattcagtttctcatcttccagtacgcctaccccatcatgcagtcgtgtctggctctgttcttccagcccaagctcgattatgccaacatctccgagcgcctcatgaagttggcctccgtgtctatgatggtctggctcattggattctacgctttcttccagaacggtctcaatcttattgccgagctcacctgttttggaaacagaaccttctaccagcagtggtggaattcccgctccattggccagtactggactctatggaacaagccagtcaaccagtactttagacaccacgtctacgtgcctcttctcgctcggggcatgtcgcggttcaatgcgtcggtggtggttttctttttctccgccgtcatccatgaactgcttgtcggcatccccactcacaacatcatcggagccgccttcttcggcatgatgtcgcaggtgcctctgatcatggctactgagaaccttcagcatattaactcctctctgggccccttccttggcaactgtgcattctggttcacctttttcctgggacaacccacttgtgcattcctttattatctggcttacaactacaagcagaaccagtag

**Figure S5**: Sequencing data of the Δ*pox* Δ*dgat*1 Δ*dgat*2. DGAT1 sequencing was performed with the primers DGAT1-Verif-F and DGAT1-Verif-R. DGAT2 sequencing was performed with the primers DGAT2-Verif-F2 and DGAT2-Verif-R2. The wild type sequence of DGAT1 and DGAT2 is given and the parts highlighted in yellow correspond to the deletion in the Δ*pox* Δ*dgat*1 Δ*dgat*2 strain.

| **Primer name** | **Sequence 5’ - 3’** |
| --- | --- |
| Elo1_P1 | ccacataaaataaagagtgttgggata |
| Elo1_P2 | gctacgaaaagactctcaacaggat |
| Elo1_T1 | cgtttttctctcctgctccc |
| Elo1_T2 | tcgccatcaccaccgc |
| ELO2_P | ctgtattctatgttacctctgctgg |
| ELO2_T | ggtgtactatcgtacattgcacc |
| RemSapI_Leu2_For | cgccgatatcggaggTtcttcctccacctc |
| RemSapI_Leu2_Rev | cctccgatatcggcggtagt |
| Oligonucleotides for pCg58 religation to pg7 | |
| PL_For | cgcgtggatccggtaccactagtgtac |
| PL_Rev | actagtggtaccggatcca |
| Oligonucleotides for target insertion in pCg58 and pg7 plasmids | |
| ELO2g1_For | GCAtgatcacaggtgcaaatcc |
| ELO2g1_Rev | AACggatttgcacctgtgatca |
| ELO2g2_For | GCAagtagaagtacatgacaacg |
| ELO2g2_rev | AACagtagaagtacatgacaacg |
| DGAT1g1_For | GCAgagtaatggtgtcgatagcg |
| DGAT1g1_Rev | AACcgctatcgacaccattactc |
| DGAT2g2_For | GCAgtcgcggttcaatgcgtcgg |
| DGAT2g2_Rev | AAccgacgcattgaaccgcgac |
| DGAT1-Verif-F | gctctgcaacagttctcacg |
| DGAT1-Verif-R | attgtcgacagtgtcaaggctg |
| DGAT2-Verif-F2 | gttcaagaccactgacaagcgc |
| DGAT2-Verif-R2 | acctccttctgctacacaccag |
| Tef_F | ccaccgtccccgaattacctttcc |
| Lip_R | cggatgactaactctccagagcg |

**Table S1**: Primers and oligonucleotides used in this study

| ELO1 PLT cassette – sequence 5’- 3’ |
| --- |
| ccacataaaataaagagtgttgggatattaatacagtatattgagtaattggttaattaagaactgttggattttactggattgaaacctagaggcgacccttgcgagactccaaggcaccacaaaactcttctctggggccgcatgacgcagattagatgaggtccacgttgttcatgccagggccagtcgcttttggtctctttctagccagtctggacccataagccatttggacaatacaatttaatttaatccagcacaaactctccgataagtcgatctctggcgaagatttacaaggcttgagaggttttatgtttcgtattgcacgcgagtgggttggtctggtgtttttccgattgcaattgattttctgtcatttgttctcggttttcctattgattttatccatcgcccaatttcttttattttacccacctatgtttaatattgtatattttgctccatagtttcttctctatcaagaataccccgctgtctcaaaccctctgcactttcccaacaagtttaatgggtagcggagatatatcctgaggatgtggggcatggtgcatgtgaattccttgcgaggtttcgatgttttcctccaattttgttctcgaattcccgtttctccgtttctccaaatgtgttgagctcatgcatttgctcatgcaatgtcgtgtgacttgtatggggaaaagataatctgtcaagttggtggtatttgtatttgtatttgtatttgttcgggggagaccgaaagagcatccatgttgactcagattgagaccaaatatctggtacaaacatctctaatcactagcgtttgaatcccatgtcccgtagtggttgtagagagctggcctgcccgttttccatcaaagtctagagtgcatatttcatgcatgtggggagcaactcgttaattttaacagcttgggtgaaaaatgccgcaagaaaaagcaggaaaaaactcactaaagcggtctcccctgtaaaccacaagaggagagtcgcataaacacctctgtttctcgcggcaacgttagaattgcctcggatccgggccgcacactcccaccaacacgcacgattttctcagattctaaatgaatatttctatttggaaaggctcacctgttgaactcaacaaggatgcaggcacgtcaacaaaaacagtagtggcggaagctggtaaccgagaccggggcaacgaggatgaaggaggggcgggagaagaattctgagggtggaataatactcgcacgaccacgtggaatacaccggcggttttattccgtcaaaaccccgcggttcagagtttctacgactgtccctcggcttaactccgtccactttttggtttctgtttgagtttgaattacgattgaaaatgcgaatttgacggtgaaaattgtcggaatgcgacgatagaaacactggaatcgacaaaactgcctgctccggaggagatgaaacactcaaaaatcgcatatcttgcctctaaaagctccgacccgctcacccggcaaacaggtcacataacctaaaatgctaactccgaactctaaccaagtctctagatatatacataacagtgatcctgttgagagtcttttcgtagcggtagggataacagggtaattatcgcttcggataactcctgctatacgaagttatacgaattcgaatatacagtaacaagctaccaccacactcgttgggtgcagtcgccagcttaaagatatctatccacatcagccacaactcccttcctttaataaaccgactacacccttggctattgaggttatgagtgaatatactgtagacaagacactttcaagaagactgtttccaaaacgtaccactgtcctccactacaaacacacccaatctgcttcttctagtcaaggttgctacaccggtaaattataaatcatcatttcattagcagggcagggccctttttatagagtcttatacactagcagatcctgccggtagaccaacccgcaggcgcgtcagtttgctccttccatcaatgcgtcgtagaaacgacttactccttcttgagcagctccttgaccttgttggcaacaagtctccgacctcggaggtggaggaagagcctccgatatcggcggtagtgataccagcctcgacggactccttgacggcagcctcaacagcgtcaccggcgggcttcatgttaagagagaacttgagcatcatggcggcagacagaatggtggcaatggggttgaccttctgcttgccgagatcgggggcagatccgtgacagggctcgtacagaccgaacgcctcgttggtgtcgggcagagaagccagagaggcggagggcagcagacccagagaaccggggatgacggaggcctcgtcggagatgatatcgccaaacatgttggtggtgatgatgataccattcatcttggagggctgcttgatgaggatcatggcggccgagtcgatcagctggtggttgagctccagctgggggaactcgtccttgaggactcgggtgacagtctttcgccaaagtcgagaggaggccagcacgttggccttgtcaagagaccacacgggaagaggggggttgtgctgaagggccaggaaggcggccattcgggcaattcgctcaacctcaggaacggagtaggtctcggtgtcggaagcgacgccagatccgtcatcctcctttcgctctccaaagtagatacctccgacgagctctcggacaatgatgaagtcggtgccctcaacgtttcggatgggggagagatcggcgagcttgggcgacagcagctggcagggtcgcaggttggcgtacaggttcaggtcctttcgcagcttgaggagaccctgctcgggtcgcacgtcggttcgtccgtcgggagtggtccatacggtgttggcagcgcctccgacagcaccgagcataatagagtcagcctttcggcagatgtcgagagtagcgtcggtgatgggctcgccctccttctcaatggcagctcctccaatgagtcggtcctcgaacacaaactcggtgccggaggcctcagcaacagacttgagcaccttgacggcctcggcaatcacctcggggccacagaagtcgccgccgagaagaacaatcttcttggagtcagtcttggtcttcttagtttcgggttccattgtggatgtgtgtggttgtatgtgtgatgtggtgtgtggagtgaaaatctgtggctggcaaacgctcttgtatatatacgcacttttgcccgtgctatgtggaagactaaacctccgaagattgtgactcaggtagtgcggtatcggctagggacccaaaccttgtcgatgccgatagcgctatcgaacgtacccagccggccgggagtatgtcggaggggacatacgagatcgtcaagggtttgtggccaactggtaaataaatgatgactcaggcggaattcagaataacttcgtataatgtatgctatacgaagttatgtagggataacagggtaatcgcgtttttctctcctgctcccaagtcattcagacttctaacatatcacaatatacgacacttgagtctataattctactgttattttggccatgtcttgatttttgtttttaatttaattgttcctatattcgtattaaattgcattaaatgactgttaaattctccatatattctccatttaataaccattgccctattgatcctgcatttttttcttttctgcagcaatttgccgaaaacggtataactaggaatagggttacttctatatggggatgtatactgatttaaggtttaaggcgtgtgagcagtgtgggttagggtatttggagttagggttgctattttcagatgtagcgatatgatgagatgtggtatgattgaagctcagaagacgctctaaagtggatagataatcagtagggtagcttttaatggtatttaattacgatcttggtctcaggaatcttatgtaccagcttttcgaagctcccacatcaatattgtagcttttctgcaatgttttatgtcattcaacctgtatcaaaacaataaattagccaataatttctatgacgcatctaattactgtcccccattccatttaccccccatatttccccatttccgacatcaccaaatatatcccatctcatatcactcacatgtccttgaaattaactttttcagaattaacctgaatttatcatttttaaattataaaattcctttaatttaaatccactctttcagtcacctttcgtcccttcgtcatgagacagatcgtaaagacgccaaagatatgccttgattttttctccattgtctcagaaactcctccggccaaaatcgagcccgatttcaaccacaaattgtgataaatacactccacccctatgctattttctatatacactatatatatatatatacactatatttttggatgctgctgtgcaaagttcaagagcacaataaatgtatatatagacaaaggaacccacgtggtcgtgtgagtggatgatgaggcgcgtcacgtgactgcaatcgacggcggagttctattttccttcattcagagtcggtcacgtgacgccgatcatgtgtgtgttggaccaatgactaggttgttattcggttatatgtattttggggtaagggggtgtattaatttgggagataaatatctcattgtgtagctacgtactctgggagtccattggtgtattccaattatcggtagcatttatcgatcacgagatattggcgttatacctgcctacagatgacctgcatctttcgcattatcgaagggagagactaatggcaaaaagtactgataacgaatttcaggggtttacggtggttgaaaagagatttggaaaaagtacgattgtagagctcggatctggctctccagcaagctattttgctacctcccaccaccactatgacacaaattgagtgcctttggccaacggtgtgaaacacaactctaaggcgtataatacactgagagtaagaatcaagagagagtatagatagagatattgaggtgttagatagaggaggggagaacatgaggggggaaggggcggtggtgatggcg |

**Table S2**: Sequence of the PLT-ELO1 cassette designed to carry out ELO1 gene deletion

| Guide name | Sequence 5’ – 3’ | CRISPR/Cas9 plasmid |
| --- | --- | --- |
| Elo2 g1 | tgatcacaggtgcaaatcc | pCg58-ELO2g1 |
| Elo2 g2 | agtagaagtacatgacaacg | pg7-ELO2g2 |
| DGAT1 g1 | gagtaatggtgtcgatagcg | pCg58-DAGT1g1 |
| DGAT2 g2 | ccgacgcattgaaccgcgac | pg7-DAGT2g1 |

**Table S3**: Sequence of the guides used to carry out deletion of ELO2 gene or DGAT genes using CRISPR-Cas9 tool.

Standard molecular biology protocols were applied for basic cloning. Plasmid digestions for constructions were typically performed using commercial restriction and modification enzymes (NEB), and DNA fragments of interest were gel purified using a gel extraction kit (Sigma). Ligations were typically carried out at 21°C for 2 h with T4 DNA ligase, or alternatively with Quick Ligase for 5 min. First, the LEU2 gene of a pCg22 (unpublished data) was mutated to remove a SapI restriction site (see PCR conditions below). After this, a fragment containing the modified marker was excised from the resulting plasmid by AatII/BamHI restriction digestion and ligated in a generic pCg50 vector digested with the same enzymes, yielding the generic plasmid pCg52. The ScR1 sequence fragment was then retrieved by digestion with KpnI and AvrII of a pCg54 (unpublished data) generic plasmid and ligated in pCg52 between the two sites, giving the generic pCg57 plasmid. Finally, a smaller version of this plasmid named pg7 was generated by removing the Cas9. To this effect, the fragment was removed by MluI/KpnI digestion, the vector band was gel purified and self-religated using small compatibles adaptators oligonucleotides (see table 1). Target sequences were introduced in the generic plasmids yielding pCg58-ELO2g1, pCg58-DGAT1g1, pg7-ELO2g2, and pg7-DGAT2g2.

**Method S1:** plasmids construction for CRISPR/Cas9 genome editing.
